# Supplementary material for: Proinflammatory pathways contribute to the pathogenesis of Clostridioides difficile infection in a murine model using Spatial transcriptomics
Source: Sci Rep. 2025 Oct 8;15:35141. doi: 10.1038/s41598-025-19106-3 (PMC12508427; doi:10.1038/s41598-025-19106-3)
Supplement: Supplementary file 2 — Supplementary Material 2 [file 41598_2025_19106_MOESM2_ESM.docx]

**Supplementary Table 1:**
Raw data used to generate Figure 3, comparing gene expression between symptomatic and asymptomatic mice.

**Supplementary Table 2:**
Raw data used for generating Figure 4, comparing gene expression profiles between superficial colonic regions of symptomatic and asymptomatic mice.

**Supplementary Table 3:**

Raw data used for generating Figure 5, comparing gene expression profiles between deep colonic regions of symptomatic and asymptomatic mice.

**Supplementary Table 4:**
Raw data used for generating Figure 6, comparing gene expression between superficial and deep colonic regions in symptomatic mice.

**Supplementary Table 5:**
Raw data used for generating Figure 7, comparing gene expression between superficial and deep colonic regions in asymptomatic mice.

**Supplementary Table 6:**
Pathway enrichment analysis results corresponding to Figure 9. This dataset includes significantly enriched pathways derived from differentially expressed genes across colonic regions.
